# An Abnormal Presentation of Multiple Myeloma in Pregnancy: A Case Report

**DOI:** 10.7759/cureus.23363

**Published:** 2022-03-21

**Authors:** Robert M Chory, Ryan Cone, Joshua Hollingsworth

**Affiliations:** 1 Obstetrics and Gynecology, Edward Via College of Osteopathic Medicine-Auburn, Auburn, USA; 2 Pharmacology, Edward Via College of Osteopathic Medicine-Auburn, Auburn, USA

**Keywords:** serum protein electrophoresis (spep), obst gynec, hematologic malignancies (hm), and pregnancy, diagnosis of multiple myeloma

## Abstract

Multiple myeloma (MM) is an uncommon hematologic malignancy in the general population and extremely rare in pregnant and peripartum women. Here we report a rare case of multiple myeloma in a 43-year-old African American woman at 24 weeks gestation who presented for severe flank pain and difficulty breathing. An elevated D-dimer was present on initial presentation and diagnoses of deep vein thrombosis and pulmonary embolism were ruled out via bilateral Doppler ultrasounds and CT scan. Lytic bone lesions, anemia, and elevated total protein were also noted and an iliac crest biopsy demonstrating monoclonal plasma cells confirmed the diagnosis of MM. She was started on steroids at 27 weeks gestation and delivered via c-section at 30 weeks gestation. Postpartum, a treatment regimen to allow for breastfeeding was discussed but was unable to be accommodated, and she was started on a multi-drug chemotherapy regimen. There have only been about 30 cases of multiple myeloma in pregnant women reported in the literature. The rarity and lack of information on the effects of multiple myeloma in pregnancy was the primary indication for publication of this case.

## Introduction

Multiple myeloma (MM) is a malignancy originating from the clonal proliferation of neoplastic plasma cells [1]. The condition is most commonly observed in males, African Americans, and the elderly, with a median age of onset of 67-70 and only 3% of cases being diagnosed before age 40 [2]. There is only an estimated 12-37 reported cases of multiple myeloma pregnancy reported in the literature [3] and only 10% of all cases of MM occurring before age 50 [2].

Multiple myeloma is a malignancy with an estimated incidence of 7 in 100,000 [2,3]. The only known risk factors for MM are high BMI, family history of MM, and exposure to Agent Orange in patients with monoclonal gammopathy of undetermined significance [4-8]. The classic presentation of multiple myeloma in the general population includes bone pain from lytic lesions, hypercalcemia, renal injury, and normochromic, normocytic anemia which was the most common finding (present in 97% of cases of MM) [9-12].

Diagnosis is typically suspected after pathologic fracture or abnormal lab findings, especially high total protein levels. Patients suspected of having MM should receive a serum protein electrophoresis (SPEP) and serum-free light chain assay in addition to routine hematologic labs such as a complete blood count (CBC), peripheral smear, and serum chemistries. A bone marrow biopsy is typically needed for a definitive diagnosis. The diagnostic criteria for MM according to the International Myeloma Working Group requires clonal bone marrow plasma cells to be greater than 10% plus the presence of end-organ damage, classically remembered by the acronym “CRAB” (hypercalcemia, renal failure, anemia, lytic bone lesions) or the presence of biomarkers associated with inevitable end-organ damage [8].

## Case presentation

Initial presentation

A 43-year-old, G5 P1213 African American female at 24 weeks gestation presented to an outpatient clinic for routine prenatal care with a complaint of severe flank pain with no relief from non-steroidal anti-inflammatory drugs (NSAIDs) or heating/cooling topical pads. The patient admitted to pain with inspiration and ambulation but denied dysuria, hematuria, or history of trauma and was referred to labor and delivery for further evaluation. Three days after the initial encounter, the patient was admitted to the hospital for further evaluation of back and flank pain. CBC, complete metabolic panel (CMP), D-dimer, COVID-19 Ag screening, and peripheral blood smear were initially ordered. Significant results of the aforementioned tests included a red blood cell (RBC) count of 3.08 (reference range of 4.00-5.20 M/CMM), hematocrit of 28% (reference range of 36-46%), hemoglobin of 9.3 (reference range 12.0-15.5 g/dl) albumin of 2.5 (reference range 3.5-5.7g/dl), sodium of 129 (reference range of 136-145 mmol/L), total protein of 9.4 (reference range of 5.8-7.9 g/dl), D-dimer 1.91 (reference range 0.19-0.49 mg/L).

Of note, calcium levels were within normal limits (8.9 with a reference range of 8.6-10.3) and GFR was also within normal limits (87 with a reference range of greater than 60), and a negative COVID-19 result was also present.

Exclusion of pulmonary embolism or deep vein thrombosis was indicated due to the abnormal D-dimer in addition to a low to moderate pre-test probability using Well’s criteria.

**Figure 1:**
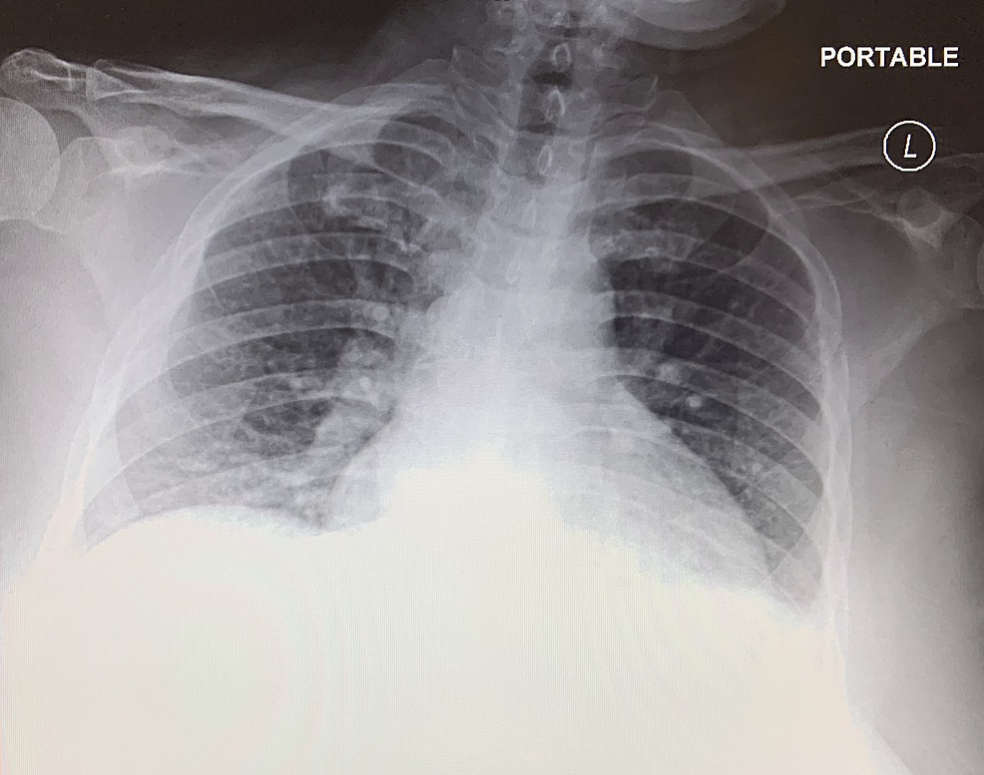
X-ray demonstrating decreased lung volumes with central bronchovascular crowding and bibasilar sub-segmental atelectasis in addition to several indeterminate lytic lesions of the ribs bilaterally.

A chest X-ray was also ordered due to a complaint of side pain. Radiology interpretation of the chest X-ray indicated low lung volumes with central bronchovascular crowding and bibasilar sub-segmental atelectasis in addition to several indeterminate lytic lesions of the ribs bilaterally, as seen in Figure 1.

Day 1 post-admission

Based on this initial set of labs and imaging, further evaluation was needed and a computed tomography angiography (CTA) of the chest with and without contrast was ordered along with a serum beta-2-microglobulin, uric acid level, serum protein electrophoresis (SPE), total protein, and urinalysis with microscopy.

Immunofixation demonstrated monoclonal IgG with kappa light chains. SPE demonstrated elevated alpha-2-globulin (0.86 with a reference range of 0.42-0.85gm/dl), elevated gamma globulin (5.15 with a reference range of 0.60-1.40 gm/dl) while beta globulin and alpha-1-globulin levels were both within normal values. Total protein was also elevated at 9.5 (reference of 6.4 - 8.2 gm/dl). Urinalysis demonstrated 1+ protein with 3-5 RBC per high-powered field with no presence of nitrites or ketones.

The CT angiogram (CTA) demonstrated no evidence of pulmonary embolism, aortic dissection, aortic aneurysm, mediastinal masses, or hilar lymphadenopathy. Significant findings included trace pleural effusions, an expansile destructive lesion of the right scapula, fracture of the right lateral fourth rib and left lateral fourth rib, multiple lucent vertebral body lesions, and a lytic lesion in the sternum. A follow-up MRI was suggested by radiology.

Day 2 post-admission

**Figure 2:**
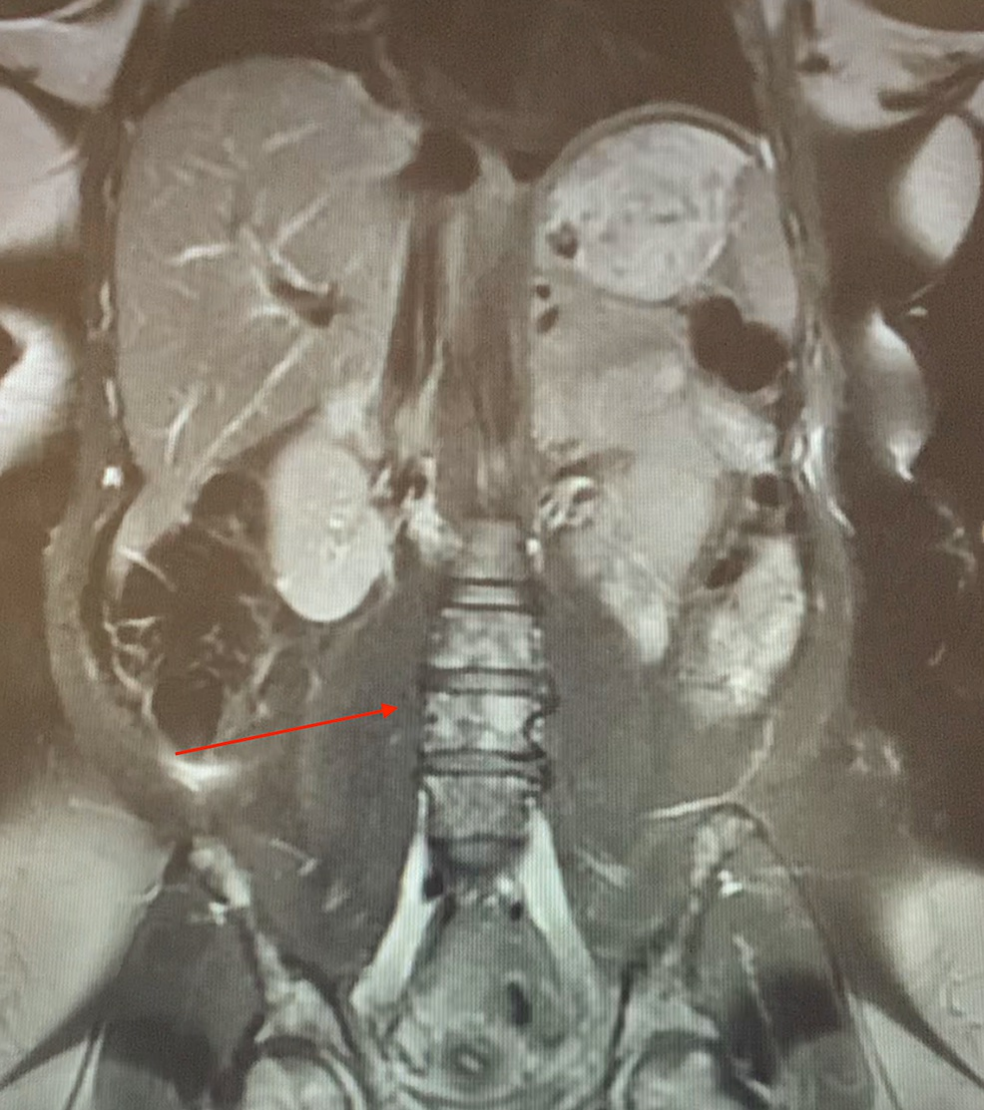
MRI of the lumbar spine without contrast showing diffuse lytic lesions (red arrow).

The follow-up CBC and BMP were obtained in addition to serum protein electrophoresis, immunoglobulin free light chain, immunofixation electrophoresis, immunoelectrophoresis Quant B, carcinoembryonic antigen (CEA), CA-125, CA-19-9, alpha-fetoprotein (AFP), total protein, IgA, IgG, and IgM level. All values were within normal limits with the exception of an extremely elevated IgG level, elevated gamma globulin, elevated random urinary protein, elevated kappa free light chains, and an elevated CEA. An MRI of the abdomen without contrast and an MRI of the thoracic spine without contrast was performed and demonstrated a heterogenous marrow signal throughout the spine and pelvis, indicating lytic osseous lesions correlating to the same areas visualized on the previously performed CTA, as shown in Figure 2. Oncology was consulted and suggested an iliac crest bone marrow biopsy to differentiate between metastatic disease and multiple myeloma.

Day 3 post-admission

Left iliac crest biopsy results demonstrated a plasma cell neoplasm with plasma cells, including atypical forms, present in over 60% of nucleated marrow cells with several areas showing sheets of plasma cells, confirming the diagnosis of multiple myeloma.

Post-discharge

The patient was discharged with instructions to follow up with her obstetrics and gynecology and oncology physicians to determine a treatment plan after delivery. She was started on steroids at 27.4 weeks with a scheduled c-section at 30 weeks gestation. She had an uncomplicated caesarian delivery. After delivery, she was given one week for recovery and then started on a multi-drug chemotherapy regimen. Notably, the patient was interested in starting a chemotherapy regimen that would still allow her to breastfeed, however, according to oncology this was not possible due to the teratogen effects of the medication regimen.

## Discussion

Diagnosis of multiple myeloma during pregnancy or in the peripartum period is very rare and there are currently few studies describing the two conditions occurring together. The clinical presentation of MM in pregnancy can vary greatly, posing a challenge for diagnosis. There appears to be no correlation between the time of diagnosis and the stage of pregnancy, however, the majority of patients were determined to be in stage 3 on initial presentation, according to the Durie-Salmon staging system [2]. Clinical findings of MM in pregnancy also contribute to the difficulty of diagnosis are the most common symptoms of MM such as proteinuria, fatigue, diffuse joint pain, and shortness of breath can all be attributed to normal pregnancy and may be overlooked during routine management [1,8,13]. Abnormal initial presentations have also been reported by Jurczyszyn et al. and Bouzguenda et al. who describe a pregnant patient who presented for pneumonia and breast lumps on initial presentation respectively, but were later diagnosed with MM [2]. Patients who present with an elevated d-dimer, as seen in our case, should first have deep vein thrombosis and pulmonary embolism ruled out before testing for alternative diagnoses [13,14]. Historically, fetal and maternal outcomes in mothers diagnosed with MM while pregnant are good. Of the 44 cases of MM in pregnancy currently published, only three cases reported fetal death, and only eight cases reported maternal death shortly after delivery [2,14].

There is currently no official guideline for the management of MM in the pregnant population, but it should be noted that the goal of management is twofold. The first aim of treatment is to deliver the fetus once viable. Depending on the time of diagnosis, intrapartum steroids should be administered prior to delivery to ensure fetal lung maturity before birth. C-section should also be used as the mechanism for delivery in cases where the fetus is able to be delivered prematurely [14-16]. Ultimately, delivery should be planned as close to 37 weeks gestation as possible while still ensuring the health of the mother, however, the clinical picture will determine how feasible this is [2,14]. The second goal of treatment is the management of the malignancy itself. The current regimen used to manage MM includes hematopoietic stem cell transplant in those eligible followed by maintenance therapy. In those ineligible for stem cell transplant, maintenance therapy with lenalidomide and dexamethasone is the standard of care [15,16]. Other pharmacologic therapies reportedly used in the management of MM include cyclophosphamide, bortezomib, and carfilzomib. These therapies are highly teratogenic with cyclophosphamide classified as category D and lenalidomide category X for use in pregnant women and have been observed to cause birth defects in 25% of women treated while pregnant. Ultimately the information on diagnosis and management of MM in pregnancy is lacking, making treatment very difficult, and more information on the topic is needed [2,14,16].

## Conclusions

Multiple myeloma is a relatively uncommon plasma cell malignancy in the general population, but extremely rare in pregnancy and in those under age 50, making this case unique. She presented with anemia and lytic bone lesions; however, it should be noted that the patient’s anemia was likely dilutional anemia associated with normal pregnancy and cannot be solely attributed to this condition. Ultimately, available information on the diagnosis and management of MM in pregnancy is inadequate, making treatment very difficult; therefore, more studies on the topic are required. 
